# Are all MVPA minutes equal? Associations between MVPA characteristics, independent of duration, and childhood adiposity

**DOI:** 10.1186/s12889-021-11420-5

**Published:** 2021-07-06

**Authors:** Aaron Miatke, Carol Maher, François Fraysse, Dot Dumuid, Tim Olds

**Affiliations:** 1 Alliance for Research in Exercise, Nutrition & Activity (ARENA), Allied Health and Human Performance, University of South Australia, Adelaide, Australia; 2 Present address: Centre for Adolescent Health, Level 5, Murdoch Children’s Research Centre, Royal Children’s Hospital, 50 Flemington Road, Parkville, 3052 Australia

**Keywords:** MVPA, Obesity, Overweight, Accelerometry, Lifestyle

## Abstract

**Background:**

The inverse relationship between moderate-to-vigorous physical activity (MVPA) duration and childhood adiposity is well established. Less is known about how characteristics of MVPA accumulation may be associated with adiposity, independent of MVPA duration. This study aimed to investigate how the MVPA characteristics of children, other than duration (bout length, time of day, day-to-day consistency, intensity), were associated with adiposity.

**Methods:**

Cross-sectional study of the Australian arm of the International Study of Childhood Obesity, Lifestyle and the Environment (ISCOLE) (participants: *n* = 424, age range 9–11, 44% male). Adiposity was determined by percent body fat via bioelectrical impedance. MVPA duration and characteristics (bout length, time of day, consistency, intensity) were derived from 7-day, 24-h accelerometry. Generalised estimating equations were used to examine the individual and multivariate associations between MVPA characteristics and adiposity.

**Results:**

Univariate analyses showed that higher MVPA duration (β range = − 0.26,-0.15), longer bouts of MVPA (β range = 0.15,0.22) and higher MVPA intensity (β range = − 0.20,-0.13) were all inversely associated with adiposity (all *p* < 0.05). When models were adjusted for MVPA duration, only MVPA intensity (β range = − 0.16,-0.04) showed consistent significant associations with adiposity.

**Conclusions:**

Characteristics of MVPA other than duration and intensity appear to be unrelated to adiposity.

## Background

Excess childhood adiposity has been described as one of the most pressing public health challenges of the twenty-first century, with almost 25% of Australian children now classified as overweight or obese [1, 2]. The negative impact of overweight on an individual’s health is significant, with both psychosocial and physiological wellbeing affected [3]. The role that physical activity (PA) plays in weight maintenance has been studied in depth. Most epidemiological research, and current guidelines [4, 5], specifically focus on the daily duration of moderate-to-vigorous PA (MVPA), which is generally defined as activity ≥3 Metabolic Equivalents (METs) in intensity [6]. Duration of MVPA plays an important role in the prevention and treatment of overweight and obesity in children, with cross-sectional and longitudinal studies generally reporting an inverse relationship between MVPA duration and adiposity levels [7].

However, despite a well-established inverse relationship between MVPA duration and adiposity [8], the possibility that characteristics of PA accumulation, such as bout length, time of day of participation, day-to-day consistency, and intensity may independently influence adiposity is often ignored, with many studies exclusively investigating the association of fatness with overall duration of MVPA, generally defined as average daily minutes of MVPA [9, 10]. Similarly, although there are guidelines for a daily total of MVPA (≥60 min), there are no specific recommendations as to how or when it should be accumulated [5].

Despite the obvious convenience of using a single metric of average daily minutes of MVPA, this ignores the ‘how and when’ of a child’s PA participation. When we consider energy intake, there is a school of thought suggesting that ‘ … a calorie is not a calorie’ [11], referring to how meal timing and the macronutrient content of food may influence weight status, independent of total caloric intake. Similarly, researchers have recently tried to promote not only reducing total sedentary time to improve metabolic health, but also to change the pattern of accumulation by breaking up long periods of inactivity [12, 13]. It is possible that the same may be true for PA, where ‘ … a minute may not be a minute’, and that characteristics of PA accumulation may influence levels of adiposity, independent of MVPA duration. This is particularly relevant given children usually amass a daily total of MVPA through the accumulation of multiple, highly active episodes of play, intermitted with periods of rest [14]. Moreover, accelerometers now allow for the detailed description of habitual PA in terms of duration, intensity, and frequency, all in a time-stamped manner [15]. This allows researchers to construct a detailed minute-by-minute PA profile and hence offers a viable way of examining characteristics of MVPA accumulation in children, beyond a daily total [16], however few studies have investigated these characteristics as possible factors.

It is possible that these characteristics could confer small, but significant, benefits in the long term in the regulation of adiposity. For example, some argue that due to the influence of circadian rhythms on metabolism, exercise timing should be considered in the treatment of metabolic disease [17]. With evidence that fat oxidation may be higher in the afternoon than the morning, for the same volume of activity [18]. Acute physiological responses that are advantageous in weight management (fat oxidation, metabolic rate, insulin sensitivity) may also last up to 72 h after the completion of activity [19, 20], meaning that consistent daily participation could confer regular small, but significant benefits that may influence adiposity levels in the long term. Bout length may also be important. Intermittent exercise of equivalent duration and intensity has been found to result in a higher excess post-exercise oxygen consumption (EPOC) when compared to a single continuous bout [21, 22], and therefore a greater total energy expenditure. It is unknown however, whether these factors may contribute to long-term weight maintenance in children.

The aim of this study was to assess how characteristics of MVPA, specifically, bout length, time of day (early vs late in day), consistency (across days), and intensity were associated with adiposity, and whether these associations were independent of MVPA duration in 424 Australian 9–11 year olds.

## Methods

This study was a secondary analysis of data from the Australian arm of The International Study of Childhood Obesity, Lifestyle and the Environment (ISCOLE) [23]. ISCOLE was a 12 nation, cross-sectional study aimed at determining associations between lifestyle behaviours and markers of obesity. For full methodological details refer to Katzmarzyk et al. [23].

Methods were carried out in accordance with relevant guidelines and regulations. Ethics approval for ISCOLE was obtained from The Pennington Biomedical Research Center Institutional Review Board (ISCOLE coordination centre), the University of South Australia Human Research Ethics Committee (approval number: 0000024016), the South Australian Department of Education and Child Development, and the South Australian Catholic Education Office. ISCOLE is registered on ClinicalTrials.gov, Identifier: NCT01722500.

Participants were children aged 9–11 recruited from 26 schools within the urban and suburban Adelaide region. All schools around the Adelaide region including public, independent and Catholic schools were stratified into tertiles based on their socioeconomic status, determined by their Index of Community Socio-Educational Advantage (ICSEA) ranking, a government ranking system [24]. Schools were randomly chosen within each tertile and asked to participate (school participation rate 43%). If a school agreed, all grade 5 children from that school were invited to participate in the study (student participation rate 57%). Written informed parental consent was then required for a child in order to participate, as well as assent from the child [23]. In total, 525 children participated in the Australian arm of the ISCOLE study. To be included in the present study participants needed to have complete PA, adiposity and sociodemographic data, meaning the final sample size was 424 (46% male).

Objective PA measures were obtained using the Actigraph GT3X+ accelerometer [25], with a sampling rate of 80 Hz. The accelerometer was worn at the waist on the right mid-axillary line. Participants were encouraged to wear the device for the full 24-h period each day, over the 7-day monitoring period (including 2 weekend days). The resulting raw accelerometer data were then filtered using Actilife 5.6 software [26] and summed into 1-min epochs.

In the present study, periods of sleep and non-wear time were distinguished from wake, wear time using a previously published algorithm developed from the ISCOLE dataset [27] which is shown to not differ significantly from expert visual inspection. Cut-points published by Evenson et al. [28] were then used to distinguish all waking, wear-time hours into intensity classifications (i.e., sedentary ≤100 cpm; light 101–2295 cpm; MVPA ≥2296 cpm). For a participant’s data to be considered valid they needed to have at least four valid monitoring days, inclusive of at least one weekend day. For a day to be valid it needed at least 10 h of waking wear time.

Average MVPA duration, time of day, and intensity values were calculated for weekdays and weekends, and then weighted 5:2 to calculate a weekly average. This accounted for differences in the way children participate in PA from weekdays to weekend days [29], and the fact not every child had the same amount/type of valid monitoring days.

Bout length was quantified using the alpha (α) value describing the relationship between bout duration and bout frequency, with a minimum bout duration of one minute. Alpha was calculated using the method proposed by Chastin and colleagues to quantify the fragmentation of sedentary bouts [30] and comes from the observation that the relationship between bout duration and bout frequency follows a power law, i.e. people participate in a high number of relatively short bouts, but fewer, relatively longer bouts contribute a higher proportion of overall MVPA duration. A higher α value indicates more fragmented MVPA accumulation patterns, and a tendency to participate in shorter duration bouts to accumulate their weekly total. On the contrary, a lower α value indicates that MVPA tends to be accumulated in relatively fewer, long bouts [31]. Importantly, α is in principle independent of total duration of MVPA.

Time of day was operationalised using T_50%_, the time of day at which half of all MVPA was accumulated, or the daily midpoint of MVPA accumulation. That is, the point at which half of all MVPA was accumulated before this time, and half of MVPA was accumulated after this time. This gives an indication of the propensity of a participant to accumulate the majority of their PA at relatively earlier or later times throughout the day. The T50% value therefore gives us an indication of whether the bulk of a child’s total daily MVPA occurring relatively early or late in the day, independent on the total duration of MVPA.

Consistency of MVPA participation referred to how much daily variability occurred in MVPA accumulation in order to amass a weekly total. This was operationalised using the coefficient of variation of minutes of MVPA across all valid days. Because the coefficient of variation value itself describes inconsistency rather than consistency, i.e. a higher coefficient of variation denotes more inconsistent participation, the values were reverse coded in the presentation of results for ease of interpretation [32].

MVPA intensity was operationalised using the mean counts per minute (CPM) value of all MVPA, weighting weekdays and weekend-days 5:2 as described earlier.

Adiposity was operationalised as percent body fat determined via bioelectrical impedance using a portable Tanita SC-240 Body Composition Analyser [33], which has demonstrated acceptable accuracy when compared to dual-energy X-ray absorptiometry in children [34] (average absolute error + 3.9%). The covariates included socio-economic status (SES), sleep duration, maturity offset, and sex. SES was determined by highest category of educational attainment by either parent, with categories: <high school; some high school; completed high school; some post-secondary (e.g., vocational diploma or certificate); bachelor degree; postgraduate. Sleep time was measured using the same Actigraph GT3X + accelerometer used to measure PA data. The algorithm developed by Tudor-Locke et al. [27], was then used to distinguish periods of sleep time from awake time. Maturity offset was measured by a method described by Mirwald et al. [35] which predicts years from peak velocity based on anthropometric measures. Other covariates considered were age, and dietary patterns as determined by a previously published study using the ISCOLE dataset that was assessed using a 23 item food frequency questionnaire [36], however neither of these covariates showed a significant correlation with the adiposity outcomes so were excluded from further analysis for simplicity of the model.

All statistical analyses were undertaken using IBM SPSS Statistics version 23.0. MVPA intensity values were positively skewed and therefore were transformed by taking the reciprocal value [37]. Simple correlations were first investigated. Multivariate relationships between each of the MVPA characteristics and adiposity were then assessed using a series of generalised estimating equations (i.e. five equations), a type of multilevel model which accounts for the heterogeneity in the sample (students were sampled in clusters at the school level). The possibility of multicollinearity was assessed by inspection of VIF values, with all values < 3.0, suggesting multicollinearity was not problematic. **Model 1:** included all sociodemographic covariates (SES, sleep duration, sex, maturity offset), along with each MVPA characteristic individually. **Model 2**: included measures from Model 1, with the addition of MVPA duration (i.e. SES, sleep duration, sex, maturity offset, MVPA duration, and each MVPA characteristic individually).

As the transformed measures used to represent MVPA bout length, intensity and consistency have no intuitive meaning, standardised betas have been used. Based on the number of predictors specified [5], a sample of 138 participants was needed to detect an effect size of 0.15, with an alpha of 0.05 and a power of 0.80. Therefore, this study was adequately powered.

## Results

Of the 525 participants in ISCOLE (Australia), 424 (81%) provided complete PA, adiposity, and sociodemographic data, and were therefore included in analysis. Participants who were excluded from analysis were similar to those who provided complete data in all sociodemographic and adiposity measures. Although a minimum of four valid monitoring days were required for a participant’s data to be considered valid, 320 (75%) provided the full 7 days of accelerometer data. Descriptive data are displayed in Table 1.

**Table 1:** Descriptive data (means, SDs) for sociodemographic, adiposity and PA data

Demographics	Age (years)		10.3 (0.6)
	Parental education (*n*)	<high school	2 (0.01%)
		Some high school	40 (9.4%)
		Completed high school	40 (9.4%)
		Some post-secondary	157 (37.0%)
		Bachelor degree	105 (24.8%)
		Postgraduate	80 (18.9%)
	Sleep duration (min/day)		565 (42)
	Maturity offset (yr)		−1.7 (0.93)
Adiposity	% body fat		21.4 (7.3)
MVPA	Duration (min/day)		56 (25)
	Bout length (α)		1.87 (0.10)
	Time of day (T_50%_) (h:min)		14:01 (1:09)
	Consistency (%)		50.1 (17.6)
	Intensity (cpm)		3734 (753)

**Duration** is average daily minutes of MVPA; **Bout length** is the α value representing the magnitude of relationship between bout duration and bout frequency (higher α = shorter bout lengths); **Time of day** is the mean time of day at which 50% of MVPA has been accumulated (higher T50% = later participation); **Consistency** is the inverse of coefficient of variation of daily MVPA minutes (higher = more consistent participation); **Intensity** is the mean CPM value of MVPA.

Values are mean (SD) of untransformed values unless indicated.

Simple correlations calculated between the five MVPA characteristics are presented in Table 2. Significant correlations were observed for six of the 10 MVPA interactions, with a general trend that children who participated in a higher total duration of MVPA per day also tended to report more consistent day-to-day participation (*r* = 0.30), which also generally occurred in longer duration bouts (*r* = − 0.76), and at a higher average intensity (*r* = 0.35). A positive correlation was also found between MVPA time of day and intensity (*r* = 0.20), meaning participants who accumulated a higher proportion of their MVPA later in the day also generally participated in MVPA that was of a higher average intensity.

**Table 2:** Correlations among various MVPA characteristics. Data shown as R-values

	Duration	Bout length	Time of day	Consistency	Intensity
Duration	•	−0.76**	0.09	0.30**	0.35**
Bout length		•	−0.03	−0.08	− 0.41**
Time of day			•	0.02	0.20**
Consistency				•	0.11*
Intensity					•

Significant correlations * < 0.05, ** < 0.01

Multivariate associations between PA variables and adiposity are shown in Tables 3 and 4. In Model 1, higher durations of MVPA (β range = − 0.26,-0.15), longer bouts of MVPA (β range = 0.15,0.22) and higher average intensities (β range = − 0.20,-0.13), were associated with lower percent body fat (*p* < 0.05).

**Table 3:** Multivariate associations between MVPA characteristics and body fat percentage

	Model 1		Model 2	
	β	***p***	β	***p***
**Duration**	− 0.19 (− 0.28,-0.11)	**0.000**		
**Bout length**	0.18 (0.10,0.27)	**0.000**	0.08 (− 0.05,0.22)	0.23
Duration			−0.14 (− 0.27,-0.00)	**0.047**
**Time of day**	−0.04 (− 0.13,0.05)	0.42	− 0.01 (− 0.09,0.07)	0.73
Duration			−0.19 (0.28,-0.11)	**0.001**
**Consistency**	−0.08 (− 0.17,0.01)	0.06	− 0.03 (− 0.11,0.05)	0.46
Duration			−0.18 (− 0.27, − 0.10)	**0.001**
**Intensity**	− 0.16 (− 0.22,-0.09)	**0.001**	− 0.10 (− 0.17,-0.03)	**0.008**
Duration			− 0.15 (− 0.24,-0.06)	**0.002**

Dependent variable: Body fat percentage, standardised beta values reported (95% confidence).

*Model 1*: adjusted for sex, SES, average sleep duration, maturity offset.

*Model 2*: adjusted for Model 1 + MVPA duration.

**Table 4:** Multivariate associations between MVPA characteristics and body fat percentage

	Girls				Boys			
	Model 1		Model 2		Model 1		Model 2	
	**β**	**p**	**β**	**p**	**β**	**P**	**β**	**p**
**Duration**	−0.26 (− 0.39,-0.12)	**0.001**			− 0.15 (− 0.25,-0.05)	**0.004**		
**Bout length**	0.15 (0.00,0.30)	**0.047**	− 0.02 (− 0.19,0.15)	0.78	0.22 (0.10,0.34)	**0.001**	0.20 (−0.01,0.41)	0.06
Duration			−0.28 (− 0.42,-0.13)	**0.001**			− 0.02 (− 0.19,0.15)	0.82
**Time of day**	0.05 (− 0.10,0.19)	0.52	0.08 (− 0.05,0.20)	0.22	− 0.14 (− 0.26,-0.03)	**0.012**	− 0.13 (− 0.23,-0.02)	**0.02**
Duration			− 0.27 (− 0.40,− 0.14)	**0.001**			-0.14 (− 0.22,-0.05)	**0.003**
**Consistency**	− 0.07 (− 0.18,0.04)	0.20	−0.02 (− 0.13,0.10)	0.78	−0.09 (− 0.22,0.03)	0.15	−0.04 (− 0.17,0.08)	0.50
Duration			−0.25 (− 0.38,-0.12)	**0.001**			−0.14 (− 0.24,-0.04)	**0.009**
**Intensity**	−0.13 (− 0.24,-0.02)	**0.026**	−0.04 (− 0.15,0.07)	0.47	−0.20 (− 0.33,-0.07)	**0.002**	−0.16 (− 0.29,-0.03)	**0.016**
Duration			−0.23 (− 0.35,-0.11)	**0.001**			−0.10 (− 0.21,0.01)	0.07

Dependent variable: Body fat percentage, standardised beta values reported (95% confidence).

*Model 1*: adjusted for SES, average sleep duration, maturity offset.

*Model 2*: adjusted for Model 1 + MVPA duration.

In Model 2 (Model 1 + MVPA duration) associations remained strong between higher duration of MVPA and lower adiposity after accounting for accumulation characteristics, with significant associations observed in 10/12 models (β range = − 0.28, − 0.02). The negative associations between MVPA intensity and adiposity remained evident, albeit attenuated, after controlling for MVPA duration (β range = − 0.16, − 0.04) suggesting that higher intensity MVPA was associated with lower adiposity levels, independent of total MVPA minutes. All other accumulation characteristics (bout length, time of day and consistency) were generally not statistically significant in the final adjusted models.

## Discussion

The aim of this study was to investigate characteristics of MVPA and how these were associated with adiposity in children. In univariate analyses, participation in longer bouts and at a higher intensity, were significantly, and inversely, associated with adiposity. However, when duration of MVPA was jointly considered with characteristics of accumulation, MVPA duration was the most significant predictor of adiposity in children, followed by MVPA intensity, with other characteristics not associated with adiposity. Correlations among PA characteristics suggest that children who accumulate more MVPA generally do so by following similar patterns of behaviours, i.e. participating in longer bouts, more consistently each day, and at average higher intensities (Table 2).

The finding that MVPA duration and intensity are inversely associated with adiposity is consistent with previous research [7, 38]. It is also intuitive that these characteristics displayed the strongest association with adiposity, considering they both directly result in a greater energy expenditure, and therefore would be beneficial from an energy balance perspective [39]. In the present study, one extra hour of MVPA per day was associated with 3.4% lower body fat percentage after controlling for covariates.

MVPA intensity was the only accumulation characteristic to show a consistent association with adiposity independent of duration. This is consistent with previous studies which have shown VPA to be more strongly associated with adiposity than MPA [38], with one study suggesting 45 min of MPA is equivalent to 15 min of VPA in seeing favourable body composition outcomes with similar aged children [40]. However, there can be substantial variation in average intensity within MPA and VPA categories. In the current study when MVPA duration and intensity were jointly considered, the standardised beta value was higher in for duration (− 0.18) than for intensity (− 0.11). It must also be said that while MVPA, which is the focus of the current study, is important, there may also be benefits from participating in lighter intensity activity [41], which is important given this constitutes a large part of an average child’s day [42].

None of the other characteristics displayed consistent associations with adiposity after controlling for MVPA duration. This is possibly due to the modest effect of MVPA on overall energy expenditure, meaning the additional influence of other characteristics may be negligible on adiposity levels. Any impact that these characteristics may have on energy balance could also be mediated by an associated increase in energy intake after activity [43]. Despite the diet scores available showing no correlation in the present sample, the impact of diet should not be discounted entirely given the role it plays in energy balance and the known limitations that exist in the measurement of dietary intake in children [44]. Importantly though, the findings strengthen the public health message that ‘something is better than nothing’ [45] and ‘every bit counts’ [46], meaning future PA promotion strategies should try to get children moving in any way possible. It also suggests that current guidelines, which allow for amassing a daily total of MVPA through the accumulation of multiple shorter episodes of activity, are adequate [4].

Few studies have investigated PA characteristics previously, making it difficult to draw direct comparisons. A recent review involving adults suggested greater reductions in body mass could be obtained by prescribing the same overall duration of MVPA in multiple, shorter bouts when compared with the same duration of MVPA performed in a single, continuous bout [47], potentially due to the increased EPOC associated [21]. However, others find no difference [48]. Regardless, the bout lengths employed in adult studies may not be relevant to a paediatric population. In observational studies involving children, some have suggested MVPA bout length may be independently associated with adiposity [49, 50], with MVPA accrued in bouts ≥5 min predicting overweight status independently of the associated increase in total MVPA duration. It is possible that the contrasting findings to the current study are due to how the PA bout length were operationalised. Previously an arbitrary cut off (5 min) has been used to group MVPA as either occurring in a ‘bout’ or ‘non-bout’ and then compared the significance of each group on weight status. It is possible that these longer bouts capture sustained higher-intensity activities like sports, and it is the associated increase in intensity that confers the benefit, not the bout length. The smaller and less diverse sample used in our study should also be noted.

One interesting feature of this study was the differences displayed in MVPA accumulation patterns between boys and girls, and how these patterns showed different associations with adiposity outcomes. The finding that boys are generally more active than girls is consistent with almost all previous research [51]. Interestingly however, duration of activity displayed a stronger inverse association with adiposity for girls than boys, whereas the opposite was true when considering the relationship between intensity and adiposity. Previously, studies have reported mixed results when considering gender differences in the relationship between physical activity duration and adiposity [52, 53]. Interestingly, a review suggested that negative associations are more common in boys than girls [7], but also noted that many studies did not consider differences in associations between sexes. Some of the differences may be attributable to different methods of assessing physical activity, and the age of participants used. One explanation for the results of the present study could be that because boys accumulate more MVPA than girls, total duration of activity may not distinguish between adiposity levels as much as other determinants of overweight. Another interesting difference between sexes was seen for how time of day of physical activity was associated with adiposity, with no significant relationship in girls, and an inverse association in boys, which was evident even after controlling for MVPA duration. The correlation between participation in MVPA later in the day, and higher intensity activity may go part of the way to explaining this, with the types of activity undertaken later in the day, such as after-school sports, possibly conferring additional benefits beyond the associated increase in MVPA duration. Previous research also suggests that boys are more likely to accumulate screen time after school than girls [54], which is associated with increased prevalence of overweight [55, 56], and is also sometimes accompanied with consumption of unhealthy foods [57]. Therefore, it is possible that boys participating in activity after school, not only see benefits associated with an increase in MVPA participation, but also due to this activity displacing alternative pursuits during this period, which may have a negative influence.

Strengths of this study include the objective measure of PA, and the novel techniques used to explore the richness of data provided by accelerometers. However, accelerometers have limitations, including failure to detect certain types of activities like cycling and load carrying activities, while they often have to be removed entirely for swimming and contact sports [15]. Accelerometers also have lower validity in detecting lifestyle and free-play activity compared to locomotion, which is particularly relevant with children [58]. Nonetheless, accelerometers have good reliability (intra-class coefficients 0.79–0.87) in this age group when a 4–7 day monitoring period is employed [59], as was the case with this study. Furthermore, whilst the ISCOLE study aimed for a representative sample of year 5 students in a major Australian metropolitan area, the recruitment rate for schools (43%) and individual participants (57%) were relatively low. It is also unclear whether the results can be generalised to children of other ages and from other geographic areas. The cross-sectional nature of the study doesn’t allow for any information on causation. It also means that the study may not truly reflect a participant’s habitual physical activity patterns. For example, seasonal variation in activity levels have not been considered [60]. Future research should explore relationships among more diverse samples, including adult populations, to see if the results are replicated. Considering adults are likely to display larger variability in both levels of adiposity and in the way they accumulate their PA this would be a useful investigation. Additionally, while overweight and obesity are significant public health issues, they are only one indicator of overall health. The application of the PA metrics developed in this study to other health outcomes would be of interest, particularly to some health indicators that may be impacted more acutely by PA.

## Conclusion

Taken together, the results are consistent with the accepted wisdom that MVPA is an important part of the prevention and treatment of overweight and obesity in children. Future interventions should emphasise increasing both the duration and intensity of activity. The lack of independent association between activity bout length and adiposity suggests that benefits are achieved even if total MVPA duration is accomplished through many short duration episodes of activity, and that there is great flexibility as to when MVPA is accumulated. Empirically, children who accumulate a lot of MVPA do it at a greater intensity, in longer bouts and more regularly, but neither bout structure nor regularity appear to be important for adiposity. This is a welcome finding, particularly given children’s natural propensity to intermittent play-based PA rather than long, continuous bouts of activity. Rather than complicating the public health message, our findings reinforce the message that getting children more active in any way possible, as often as possible, and with higher intensity should be the priority.

## Data Availability

The data that support the findings of this study were originally collected as part of the multinational ISCOLE study. They remain the property of the Pennington Biomedical Research Center (ISCOLE coordination centre) and are available from Peter T. Katzmarzyk (Peter.Katzmarzyk@pbrc.edu) (CI of original ISCOLE study) but restrictions apply to the availability of these data, which were used under license for the current study, and so are not publicly available. Tim Olds was an investigator of the original ISCOLE study, as well as an author of the current study. Data are available from the authors (timothy.olds@unisa.edu.au) upon reasonable request and with permission of Pennington Biomedical Research Center.

## Abbreviations

CPM: Counts Per Minute
EPOC: Excess Post-exercise Oxygen Consumption
ICSEA: Index of Community Socio-Educational Advantage
ISCOLE: International Study of Childhood Obesity, Lifestyle and Environment
METs: Metabolic Equivalents
MVPA: Moderate-to-Vigorous Physical Activity
PA: Physical Activity
SD: Standard Deviation
SES: Socio-Economic Status
